# Isocitrate-dehydrogenase-mutant lower grade glioma in elderly patients: treatment and outcome in a molecularly characterized contemporary cohort

**DOI:** 10.1007/s11060-022-04230-1

**Published:** 2023-01-17

**Authors:** P. Dao Trong, M. Gluszak, D. Reuss, A. von Deimling, A. Wick, L. König, J. Debus, C. Herold-Mende, A. Unterberg, C. Jungk

**Affiliations:** 1 Department of Neurosurgery, University Hospital Heidelberg, 69120 Heidelberg, Germany; 2 Department of Neuropathology, Institute of Pathology, University of Heidelberg, 69120 Heidelberg, Germany; 3 German Cancer Consortium (DKTK), CCU Neuropathology, German Cancer Research Center, 69120 Heidelberg, Germany; 4 Department of Neurology, University Hospital Heidelberg, 69120 Heidelberg, Germany; 5 Department of Radiation Oncology and Radiation Therapy, University Hospital Heidelberg, 69120 Heidelberg, Germany; 6 Heidelberg Institute of Radiation Oncology (HIRO), 69120 Heidelberg, Germany; 7 Department of Neurosurgery, University Hospital Heidelberg, Im Neuenheimer Feld 400, 69120 Heidelberg, Germany

**Keywords:** Lower grade glioma, Isocitrate dehydrogenase mutant glioma, Elderly, WHO classification

## Abstract

**Purpose:**

Lower-grade glioma (LGG) is rare among patients above the age of 60 (“elderly”). Previous studies reported poor outcome, likely due to the inclusion of isocitrate dehydrogenase (IDH) wildtype astrocytomas and advocated defensive surgical and adjuvant treatment. This study set out to question this paradigm analyzing a contemporary cohort of patients with IDH mutant astrocytoma and oligodendroglioma WHO grade 2 and 3.

**Methods:**

Elderly patients treated in our department for a supratentorial, hemispheric LGG between 2009 and 2019 were retrospectively analyzed for patient-, tumor- and treatment-related factors and progression-free survival (PFS) and compared to patients aged under 60. Inclusion required the availability of subtype-defining molecular data and pre- and post-operative tumor volumes.

**Results:**

207 patients were included, among those 21 elderlies (10%). PFS was comparable between elderly and younger patients (46 *vs*. 54 months; p = 0.634). Oligodendroglioma was more common in the elderly (76% *vs*. 46%; p = 0.011). Most patients underwent tumor resection (elderly: 81% *vs*. younger: 91%; p = 0.246) yielding comparable residual tumor volumes (elderly: 7.8 cm^3^; younger: 4.1 cm^3^; p = 0.137). Adjuvant treatment was administered in 76% of elderly and 61% of younger patients (p = 0.163). Uni- and multi-variate survival analyses identified a tumor crossing the midline, surgical strategy, and pre- and post-operative tumor volumes as prognostic factors.

**Conclusion:**

Elderly patients constitute a small fraction of molecularly characterized LGGs. In contrast to previous reports, favorable surgical and survival outcomes were achieved in our series comparable to those of younger patients. Thus, intensified treatment including maximal safe resection should be advocated in elderly patients whenever feasible.

**Supplementary Information:**

The online version contains supplementary material available at 10.1007/s11060-022-04230-1.

## Introduction

Lower-grade gliomas (LGG) are primary brain tumors that share mutations in the isocitrate dehydrogenase gene (IDH^mut^) [1]. They are classified by the World Health Organization (WHO) as grade 2 and 3 tumors. Compared to their wildtype counterpart (IDH^wt^), which are classified as glioblastoma IDH^wt^ [2], IDH^mut^ astrocytomas fare much better, although malignant transformation into grade 4 tumors is not uncommon [3]. Another important feature of LGG is the young age at first diagnosis. The median age of IDH^mut^ astrocytoma and IDH^mut^, 1p19q co-deleted oligodendroglioma patients is 38.1 and 45.4 years, respectively [4]. As age has univocally been shown to be an important prognostic factor, it has guided treatment decisions [5–8]. An age below 40 years along with other favorable factors is considered a positive prognostic factor and a watch and wait strategy may be pursued after resection [5]. In contrast, in patients over 40, upfront maximal safe resection followed by adjuvant chemoradiation is recommended. In 2016, the WHO classification has introduced an important shift from a pure histological towards an integrated molecular diagnosis creating a new paucity in the rare elderly LGG patient population [2, 9]. Previous studies, not stratified for IDH mutation status or other molecular markers, have tried to elucidate optimal treatment and patient outcome but most likely included IDH^wt^ glioblastomas (formerly astrocytoma IDH^wt^) which are much more common in the elderly than IDH^mut^ glioma and show a dismal prognosis [10]. This may have masked positive treatment effects in previous analyses. Conclusions as to restrain from maximized surgery or adjuvant treatment are therefore questionable [11]. In lack of a molecularly defined elderly LGG cohort, treatment recommendations cannot be adequately given. Of note, there is no clear age cut-off defining “elderly” patients in the context of LGG. The available literature shows a wide range starting from 50 years (50–60 years) [11–15]. In this study, we chose the most conservative age cut-off of 60 years to establish a representative elderly cohort. Taken together, this study aims to analyze surgical and survival outcomes in a molecularly characterized, purely IDH^mut^ cohort of elderly lower-grade glioma patients.

## Materials and methods

### Patient selection

**Fig. 1:**
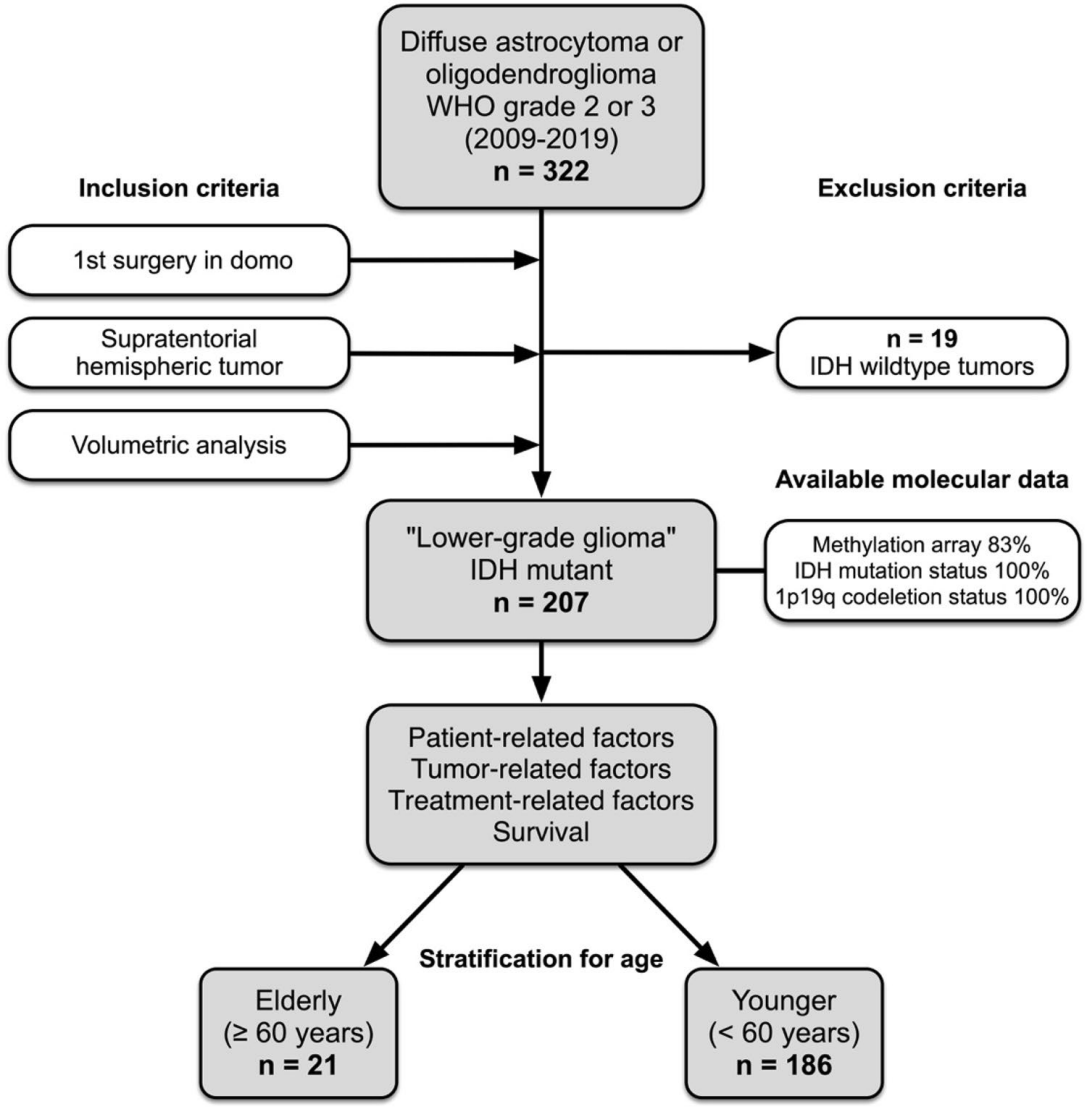
Flowchart depicting patient selection of the lower-grade glioma cohort. After applying the inclusion and exclusion criteria on 322 identified LGG patients, 207 patients were left for the final analysis. 186 patients were considered “younger” and 21 “elderly” based on the pre-specified age cut-off of 60 years

A database search was conducted for lower-grade glioma patients who were treated in our neurosurgical department from 2009 to 2019 (Fig. 1). Surgery included stereotactic or open biopsy, partial, subtotal and gross total resection. Use of surgical adjuncts such as intraoperative MRI (iMRI), 5-aminolaevulinic acid (5-ALA) and intraoperative neuromonitoring (IONM) were also recorded. For all patients, medical records were reviewed for clinical information (Karnofsky performance status (KPS), neurological symptoms). Approval from the ethics committee of the Medical Faculty of the University of Heidelberg was obtained before initiation of the study and patient consent was waived (reference S-005/2003, as of 31.01.2003).

### Histopathologic and molecular diagnosis

Neuropathologic diagnostics was performed in concordance with the WHO classification from 2016 [9]. The following molecular data were obtained as part of daily routine: IDH mutation status was obtained by immunohistochemistry or direct sequencing of the mutation hotspot region for all patients [16]. 1p/19q codeletion status was available for all patients. Genome wide methylation analysis with copy number analysis was available for 171 patients (83%) and was generated using the Illumina HumanMethylation450 (450 k) or Methylation EPIC (850 k) array platforms as described [17].

### MRI evaluation

MRI was available at initial diagnosis until a few days prior to surgery (“preoperative”). Imaging sequences included T1-, T1 + gadolinium, T2- and fluid-attenuated inversion-recovery (FLAIR)-weighted sequences. Manual segmentation was performed using the Brainlab™ software (Brainlab, Germany) to quantify tumor volumes on T1- contrast-enhanced (CE) and FLAIR-weighted images in cm^3^ at pre- and early (< 72 h) post-operative scans. Tumor features such as eloquent location, midline crossing and involvement of the subventricular zone (SVZ) were also evaluated.

### “The Cancer Genome Atlas” (TCGA) data analysis

The combined glioblastoma (GBM) and LGG cohort was analyzed using the publicly available cBioPortal platform. Only IDH^mut^ WHO grade 2 and 3 tumors were considered.

### Statistical analysis

Statistical analyses were performed using the SPSS statistics software (IBM, version 28.0.0.0) and Prism (GraphPad, version 9.4.1). Comparative statistical tests comprised Fisher’s exact test and Chi square test for categorial and Mann–Whitney-U test for continuous variables. For survival analysis, progression-free survival (PFS) was defined by the time interval from first surgery to first radiological progression, last follow-up or death, and cancer-related survival from first surgery until last follow-up or tumor-related death. A univariate log-rank test was used to identify patient-, tumor- and treatment-related variables with prognostic impact. For multivariate analysis, a Cox proportional hazard model was used with inclusion of all covariates found to be significant in univariate analysis (p < 0.05) by stepwise backward selection. Only cases with all covariates available were considered for multivariate analysis (n = 167).

## Results

### Baseline characteristics of the molecularly characterized patient cohort

**Fig. 2:**
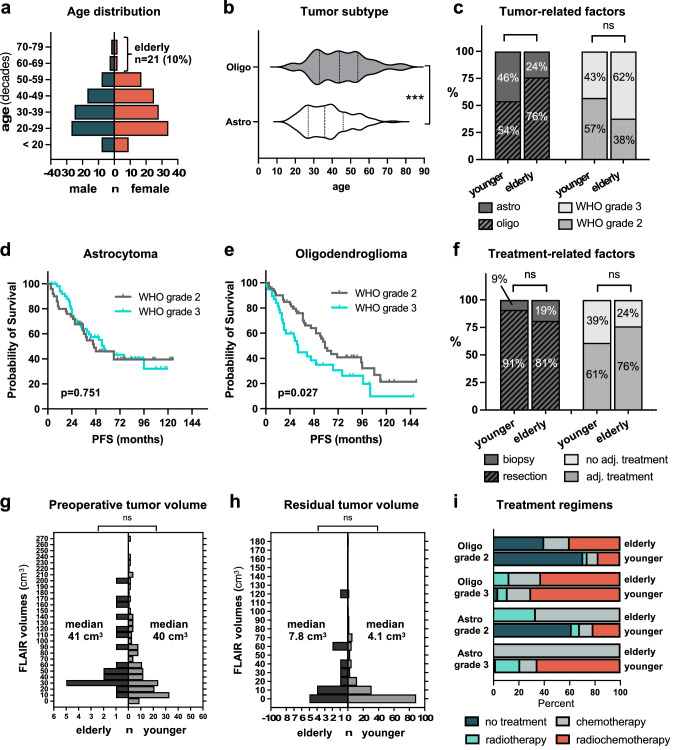
**a** Distribution of the study cohort. 21 patients (10%) were above the age of 60 and were considered elderly. Median age of the total population was 41 years (range 17–79). **b** Oligodendroglioma patients were older than astrocytoma patients (36 vs. 44 years, p < 0.001). **c** Oligodendroglioma was significantly overrepresented in the elderly (76 vs. 54%; p = 0.011), whereas WHO grade was not (p = 0.105). **d**, **e** WHO grade prognosticated PFS only in oligodendroglioma patients. **f** Surgical treatment strategies were comparable in the elderly and the younger with most patients receiving tumor resection in both cohorts (81% vs. 91%, p = 0.246). Adjuvant treatment was administered in 61% of the younger and 76% of the elderly patients (p = 0.163). **g**, **h** Preoperative and residual FLAIR tumor volumes did not differ between both age groups (p = 0.223, p = 0.137). **i** Adjuvant treatment consisted of radiotherapy or chemotherapy alone or chemoradiation and was dependent on tumor subtype. *p < 0.05, ***p < 0.001

Patients treated in our department from 2009 to 2019 were screened for a supratentorial astrocytoma or oligodendroglioma WHO grade 2 or 3 (Fig. 1). 322 consecutive patients were identified and 19 excluded (IDH^wt^ astrocytoma). Considering only patients with first surgery in our department and MRI data sets allowing volumetric analysis, the final study cohort comprised 207 patients. The median age of the study cohort was 41 years (range 17–79). 21/207 patients were over the age of 60 and were considered elderly, constituting 10% of all LGG patients (Fig. 2a). Oligodendroglioma patients were older than astrocytoma patients (36 *vs*. 44 years; p = 0.0002, Fig. 2b). Baseline patient characteristics were comparable between younger and elderly patients except for the KPS which was significantly higher in younger patients (median KPS 95 *vs*. 87; p < 0.001; Table 1). However, there were significant differences in tumor-related factors (Table 1). SVZ involvement was significantly more frequent in elderly (15/21 = 71%) than in younger patients (84/186 = 45%; p = 0.036). Also, oligodendroglioma was significantly overrepresented in the elderly (16/21 = 76% *vs*. 86/186 = 46%; p = 0.011; Fig. 2c) whereas WHO grade did not show a significant difference between the two groups (p = 0.105; Fig. 2c). The rates of tumors with contrast enhancement and those crossing the midline as well as preoperative tumor volumes (FLAIR and CE) were comparable as well (Table 1, Fig. 2g).

**Table 1:** Patient-, tumor- and treatment-related factors of the lower-grade glioma cohort stratified by age

	Elderly patients (>/= 60 years)	Patients < 60 years	p value
n	21	186	
Patient-related factors			
Gender (male:female)	10 (48%):11 (52%)	107 (58%):79 (42%)	0.488*
KPS (median)	87	95	< 0.001*
Neurological deficits (yes:no)	8 (38%):13 (62%)	41 (22%):145 (78%)	0.109*
Seizures (yes:no)	10:48%):11 (52%)	115 (62%):71 (38%)	0.243*
Incidental finding (yes:no)	3 (14%):18 (86%)	26 (14%):160 (86%)	1.0*
Tumor-related factors			
Side (right:left:bilateral)	12 (57%):9 (43%):0	85 (46%):97 (52%):4 (2%)	0.541°
Eloquent location (yes:no)	7 (33%):14 (67%)	59 (32%):127 (68%)	1.0*
Contrast enhancement (yes:no)	10 (48%):11 (52%)	70 (38%):113 (62%)	0.233*
SVZ involvment (yes:no)	15 (71%):6 (29%)	84 (45%):102 (55%)	0.036*
Multifocal lesions (yes:no)	3 (14%):18 (86%)	6 (3%):180 (97)	0.051*
Midline crossing (yes:no)	3 (14%):18 (86%)	32 (17%):154 (83%)	1.0*
T2/FLAIR mismatch sign (yes:no)	2 (10%):17 (81%)	36 (19%):147 (79%)	0.537*
Tumor subtype (astro:oligo)	5 (24%):16 (76%)	100 (54%):86 (46%)	0.011*
WHO grade (2:3)	8 (38%):13 (62%)	106 (57%):80 (43%)	0.105*
Preoperative tumor volume FLAIR median (range) [cm^3^]	41 (8.0–198)	40.3 (2.15–268.4)	0.223^&^
Preoperative tumor volume CE median (range) [cm^3^]	1 (0–44)	0 (0–112.6)	0.059^&^
Treatment-related factors			
Resection:biopsy	17 (81%):4 (19%)	169 (91%):17 (9%)	0.246*
Residual tumor volume FLAIR median (range) [cm^3^]	7.82 (0–121)	4.1 (0–180.1)	0.137^&^
Residual tumor volume CE median (range) [cm^3^]	0 (0–1.58)	0 (0–4.54)	0.44^&^
ioMRI (resected patients only) (yes:no)	n = 17, 14 (82%):3 (28%)	n = 169, 147 (87%):22 (13%)	0.596*
5-ALA (resected patients only) (yes:no)	n = 17, 0:17 (100%)	n = 169, 2 (2%):167 (99%)	0.654*
IONM (resected patients only) (yes:no)	n = 17, 1 (6%):16 (94%)	n = 169, 35 (21%):134 (79%)	0.142*
Awake craniotomy (resected patients only, yes:no)	n = 17, 2 (10%):19 (91%)	n = 169, 39 (21%):146 (79%)	0.262*
Neurological deterioration (yes:no)	2 (10%):19 (91%)	50 (27%):136 (73%)	0.111*
Adverse events (yes:no)	2 (10%):19 (91%)	16 (9%):170 (91%)	1.0*
Revision surgery (yes:no)	2 (10%):19 (91%)	14 (8%):172 (92%)	0.658*
Adjuvant treatment (yes:no)	16 (76%):5 (24%)	112 (61%):72 (39%)	0.163*
Treatment modality (none:RT:CT:RT + CT)	5 (24%):2 (10%):7 (33%):7 (33%)	72 (39%):17 (10%):22 (12%):73 (39%)	0.06°
Follow-up time (median; months)	54	70	0.272*
Progression (yes:no)	12 (57%):9 (43%)	101 (55%):84 (45%)	0.497*
PFS (median, months)	46	54	0.634*
Overall deaths (yes:no)	5 (24%):16 (76%)	17 (9%):166 (91%)	0.020*
Cancer-related deaths/non-cancer-related deaths	2:3	15:2	0.023*
Cancer-related survival (median, months)	32	39	0.638^§^

*KPS* Karnosky Performance Status, *SVZ* subventricular zone; *FLAIR* fluid-attenuated inversion-recovery; *ioMRT* intraoperative magnetic resoncance imaging; *epMRI* early-postoperative MRI; *5-ALA* 5-Aminolevolenic acid; *IONM* intraoperative neuromonitoring; *PFS* progression free survival; *RT* radiotherapy; *CT* chemotherapy; considered significant if p < 0.05

*Fisher’s exact test

^°^Chi square test

^&^Mann–Whitney test

^§^Log-rank test

### Surgical and adjuvant treatment strategies in elderly and younger patients

Surgical results were comparable in both age groups. The rates of patients undergoing resection (*vs.* biopsy) did not differ statistically in younger (91%) and elderly (81%) patients (p = 0.246; Fig. 2f). There was a trend towards a more eager use of surgical adjuncts (iMRI, IONM, awake craniotomy; Table 1) in younger patients, nonetheless yielding comparable residual volumes (median FLAIR volume 7.8 cm^3^ (elderly) *vs*. 4.1 cm^3^ (younger); p = 0.137; Fig. 2h). 34/207 patients had near complete tumor resection with residual tumor volumes < 1 cm^3^, of which 33 were younger (18%) and one elderly (5%) (p = 0.150). Importantly, the rates of postoperative neurological deterioration or revision surgery did not differ between the two groups (p = 0.111 and p = 0.658, respectively). Also, there was no significant difference in the administration of adjuvant treatment (76% elderly *vs.* 61% younger; p = 0.163). 39% of the younger patients did not receive any adjuvant treatment, mainly because of a favorable risk profile (e.g. extended resections) (Fig. 2f). In the elderly, reasoning for not administering adjuvant treatment was mixed. Of the 5 elderly patients who did not receive adjuvant treatment (24%), 3 had a favorable risk profile (oligodendroglioma WHO grade 2, gross total resection), 1 patient refused further treatment (astrocytoma WHO grade 3) and 1 patient was simultaneously diagnosed with an acute myeloid leukemia and died of its complications (astrocytoma WHO grade 3). Treatment regimens were heterogeneous and mainly dependent on tumor subtype and WHO grade. 70% of the younger oligodendroglioma WHO grade 2 patients did not receive adjuvant treatment in contrast to the elderly, where adjuvant treatment was administered in 60%. Similarly, for astrocytoma WHO grade 2 patients, 62% of the younger cohort did not receive adjuvant treatment as opposed to the elderly where all three patients received radiotherapy (n = 1) or chemotherapy (n = 2). In WHO grade 3 patients, a watch-and-wait strategy was not favored in both cohorts and only pursued in individual cases. In oligodendroglioma WHO grade 3, chemoradiation was the most frequently administered treatment regimen (younger: 70%, elderly: 63%) whereas in astrocytoma WHO grade 3 tumors only younger patients received combined treatment. For the two elderly astrocytoma WHO grade 3 patients, chemotherapy alone was administered (Fig. 2i).

### Identification of prognostic factors

**Fig. 3:**
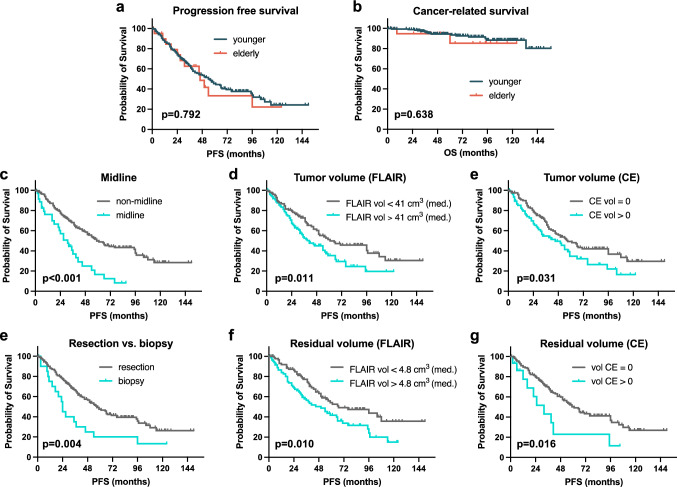
Kaplan–Meier curves depicting patient-, tumor- and treatment-related factors. Univariate analysis identified midline crossing, surgical strategy, and pre- and post-operative tumor volumes (FLAIR and CE) as prognosticators for PFS. For continuous variables (pre- and post-operative tumor volumes), the corresponding median volumes were used for dichotomization

Next, we sought to describe patient outcome and identify prognostic factors. Within a median follow-up of 54 months in elderly and 70 months in younger patients, tumor progression occurred in 57% and 55% of patients, respectively (p = 0.497). Death (due to any reason) occurred in 24% of elderly and 9% of younger patients (p = 0.02) resulting in inferior overall survival (OS) in the elderly cohort (Table 1, Suppl. Fig. 1a). However, of the 5 deceased elderly patients, 3 were not tumor-related deaths whereas 15 out of 17 younger patients died of tumor-related reasons. After censoring non-cancer-related deaths, cancer-related survival between the two cohorts did not differ (p = 0.638; Fig. 3b). For further survival analysis, we focused on PFS since time to progression and subsequent re-exposition of treatment is highly relevant in slowly progressing tumors. Importantly, PFS was comparable in elderly and younger patients (median 46 *vs.* 54 months; p = 0.792; Fig. 3a). Subsequent univariate analysis of all patient-, tumor- and treatment-related factors was performed for the complete cohort, and several factors were identified. Midline crossing conferred shorter PFS (median 31 *vs*. 59 months; p < 0.0001; Fig. 3c). Also, preoperative tumor volumes larger than the corresponding median volume (FLAIR: 41 cm^3^, CE: 0 cm^3^) were associated with inferior PFS (FLAIR: median 41 *vs*. 61 months, p = 0.01; CE: median 45 *vs*. 57 months; p = 0.034; Fig. 3d and e). Concerning treatment-related factors, patients with tumor resection had a significantly longer PFS than patients with biopsy only (57 *vs*. 24 months; p = 004; Fig. 3e). Moreover, residual tumor volumes smaller than the individual median volume (FLAIR: 4.8 cm^3^; CE: 0 cm^3^) were linked to superior PFS (FLAIR: 61 *vs*. 41 months, p = 0.01; CE: 61 *vs*. 32 months, p = 0.016). To rule out that these results were biased by patient age, we performed subgroup analysis (elderly *vs.* younger patients) for every individual prognostic factor, but results coincided (Suppl. Fig. 2). Adjuvant treatment and tumor subtype did not impact PFS (Table 2). Within the tumor subtypes, WHO grade (2 *vs.* 3) was only prognostic in oligodendroglioma patients (median PFS 59 vs. 32 months, p = 0.0274, Fig. 2d and e). Subsequent multivariate analysis with inclusion of all variables significant in univariate analysis revealed “midline crossing” as the only independent prognostic factor negatively associated with PFS (HR 2.315; 95% CI 1.39–3.85; p = 0.001; Table 2).

**Table 2:** Uni- and multi-variate survival analysis of patient-, tumor- and treatment-related factors

Variable	Univariate analysis			Multivariate analysis		
	p value	HR	95% CI	p value	HR	95% CI
Patient-related factors						
Age (elderly *vs*. younger)	0.719	1.083	0.584–2.008			
Gender (male)	0.205	1.327	0.922–1.912			
Neurological deficit (yes)	0.962	1.028	0.662–1.596			
Seizure (yes)	0.303	1.27	0.876–1.841			
Incidental finding (yes)	0.641	0.851	0.503–1.440			
KPS (> median)	0.800	0.954	0.658–1.382			
Tumor-related factors						
Side (left *vs*. right *vs.* bilateral)	0.291					
Eloquent location (yes)	0.747	1.079	0.709–1.643			
Contrast enhancement (yes)	**0.023**	1.491	1.004–2.213	0.051	1.527	0.998–2.337
SVZ involvement (yes)	0.148	1.33	0.921–1.921			
Multifocal lesions (yes)	0.663	0.8	0.356–1.798			
Midline crossing (yes)	** < 0.001**	2.468	1.378–4.420	**0.001**	2.315	1.393–3.849
T2/FLAIR mismatch sign (yes)	0.094	1.413	0.855–2.334			
Tumor subtype (astro *vs*. oligo)	0.319	0.847	0.589–1.220			
WHO grade (2 *vs*. 3)	0.336	0.82	0.568–1.184			
Preoperative tumor volume FLAIR (> median)	**0.011**	1.593	1.099–2.308	0.857	1.0	0.996–1.005
Preoperative tumor volume CE (> median)	**0.034**	1.491	1.004–2.213	0.901	0.999	0.980–1.018
Treatment-related factors						
Resection *vs.* biopsy	**0.004**	2.101	1.055–4.183	0.491	2.010	0.275–14.7
Residual tumor volume FLAIR (> median)	**0.010**	2.175	1.124–2.548	0.888	0.999	0.988–1.011
Residual tumor volume CE (> median)	**0.016**	2.175	0.874–5.413	0.408	1.164	0.813–1.666
Neurological deterioration (yes)	0.547	1.096	0.716–1.677			
Adverse event (yes)	0.555	0.783	0.382–1.607			
Revision (yes)	0.502	0.763	0.402–1.448			
Adjuvant therapy (yes)	0.319	1.692	0.558–1.204			

Results were computed using a log-rank test for univariate analysis and a Cox proportional hazard model for multivariate analysis with stepwise backward selection of all covariates significant in univariate analysis. Factors considered significant on a < 0.05 level are presented in bold

*HR* hazard ratio, *CI* confidence interval, *KPS* Karnofsky Performance Status, *CE* contrast enhancement

### Comparison with “The Cancer Genome Atlas” (TCGA) IDH mutant study cohort

To put our findings into perspective, survival data of the combined GBM and LGG cohort of TCGA was extracted [4, 18]. Only patients with a connotated age and a confirmed WHO grade 2 or 3 tumor with IDH1 (n = 358; 51%) or IDH2 (n = 19; 2.6%) mutations were included (n = 377). As in our cohort, 10% (n = 37) of patients in the TCGA were above the age of 60. Age-dependent distribution of tumor subtypes and WHO grade coincide well with our findings (Suppl. Fig. 3). Only OS, but not cancer-related survival was available for the TCGA cohort and was shorter than in our cohort (Suppl. Fig. 2a). Nevertheless, similar to our cohort, OS was inferior in elderly patients compared to younger ones (52 vs. 96 months; p < 0.0001). When looking at the tumor subtypes, OS differed among WHO grade 2 and 3 tumors in oligodendroglioma and astrocytoma patients, in contrast to our findings, where WHO grade was only relevant in oligodendroglioma patients (Suppl. Fig. 2b and Fig. 2d and e). Taken together, comparing our results with the cohort of TCGA, yielded similar results, increasing the validity of our findings.

## Discussion

This study analyzes a molecularly characterized cohort of supratentorial IDH^mut^ glioma patients with special emphasis on patients above the age of 60 (elderly) which comprised only 10% of the cohort. Younger (< 60 years) and elderly patients were comparable in most patient-, tumor- and treatment-related factors except for preoperative KPS (lower in the elderly), SVZ involvement (more frequent in the elderly) and tumor subtype (oligodendroglioma more frequent in the elderly). Midline crossing of the tumor, pre- and post-operative tumor volumes and the choice of surgical strategy influenced PFS but were independent of patient age. Importantly, treatment intensity in terms of surgical strategy, residual tumor volumes and adjuvant treatment were equally high and postoperative morbidity was equally low in both age groups, illustrating that elderly LGG patients can be treated as aggressively as younger patients as PFS did not differ.

The predicted incidence of astrocytoma and oligodendroglioma in the USA is 0.51 and 0.25 per 100,000 inhabitants per year; they account for only 6.4% of all adult primary central nervous tumors [19]. In this study cohort, only 10% of LGG patients were above the age of 60 years. Considering that about 500 primary brain tumor operations are being performed in our department each year, only three elderly LGG patients are being diagnosed per year. This circumstance makes it virtually impossible to recruit enough patients to power randomized-controlled trials (RCT) and explains the sparse literature on this rare patient population. In lack of RCTs, analysis of real-world data is a feasible alternative to understand the treatment outcomes in a broader, representative patient population as presented in this study [20].

This work focuses exclusively on IDH^mut^ astrocytoma and oligodendroglioma, the “lower-grade glioma” patients. This is of importance since IDH^wt^ astrocytoma, independent of WHO grade, has been shown to confer an unfavorable prognosis and therefore is now being classified as GBM IDH^wt^ [2, 21, 22]. Since GBM typically presents at a median age of 65, it is likely that previous studies on elderly LGG patients unintentionally included GBM patients which distorts reports on patient outcome [19]. A recent analysis by Morshed and colleagues reported on 26 WHO grade 2 glioma patients aged 60 or older. However, 26.9% harbored astrocytoma IDH^wt^ tumors, resulting in an overall PFS of 23.5 months [10]. In contrast, we report a PFS twice as long (46 months) which was also comparable to that of younger IDH^mut^ patients.

Comparing our results to the TCGA cohort, we found coinciding distributions of tumor subtypes and WHO grades in the younger and elderly undermining the validity of our findings. Interestingly, in contrast to the TCGA cohort, OS did not differ between astrocytomas WHO grade 2 and 3 in our study. According to a recent review by Von Deimling et al. analyzing studies conducted pre and post 2016, this circumstance can be ascribed to changes in classification following the 2016 CNS WHO update, while the criteria for grading remained largely untouched leading to a striking reduction of prognostic significance of diffuse astrocytic glioma grading which was also described by Olar et al. [23, 24].

In this analysis, elderly and younger patients differed only in few aspects. Interestingly, SVZ involvement was more frequent in elderly patients, a finding which hasn’t been described before. In GBM IDH^wt^, SVZ involvement has been linked to inferior survival and distinct growth and recurrence patterns [25]. In LGG patients, however, reports on SVZ involvement are sparse and warrant further investigations. While both age groups did not differ with respect to WHO grade, oligodendroglioma was more frequent in the elderly. This has been reported before and may help to explain the favorable outcome in this age population [26].

Not surprisingly, the median KPS of elderly patients was 8 points below the younger. Despite this small but measurable difference in functional status, treatment was not restrained in the elderly, and KPS per se did not affect PFS. Unlike in glioblastoma IDH^wt^ where treatment recommendations differ considerably in younger and elderly (> 65 years) patients, there are no evidence-based treatment recommendations in elderly LGG patients [27–29]. Since the initial report by Pignatti et al., a patients’ age above 40 has been considered “high risk” for poor outcome and has therefore been incorporated as a stratification factor in large-scale RCTs and evidence-based treatment recommendations [1, 5–7]. This may in part explain why adjuvant treatment was frequently administered in our younger patient cohort as well (61% as compared to 76% in the elderly).

In our cohort, surgical strategy was independent of age. Most patients underwent tumor resection (81% of the elderly *vs*. 91% of the younger patients) with comparable residual FLAIR and CE tumor volumes and low surgical morbidity in both age groups, demonstrating that maximized resection can be safely pursued in the elderly as well. This contrasts with a previous report by Youland et al. reporting on their 50-year experience on patients over 55 years harboring a WHO grade 2 glioma where resection was only performed in 36% of cases [11]. This reflects different treatment strategies which, of course, may have changed over time. Also, recent studies report a more favorable “resectability” of IDH mutant tumors compared to historical IDHwt WHO “grade 2” tumors [30, 31]. In univariate survival analysis, tumor resection as well as smaller residual tumor volumes were identified as prognostic factors for prolonged PFS, both in the overall cohort and in subgroup analysis, undermining the prognostic relevance of tumor resection in LGG patients [32, 33].

As with all retrospective analyses, there are several limitations. Treatment decisions were uncontrolled and may have biased our results. Also, the imbalance between the much larger younger and the small elderly cohort (186 *vs*. 21 patients) may affect statistical analysis and mask effects otherwise detected. Nevertheless, considering the very low incidence of elderly LGG patients, analysis of real-world data appears feasible to unravel patient characteristics and treatment outcomes.

Taken together, PFS and cancer-related survival were comparable in elderly and younger patients, owing to the exclusion of IDH^wt^ tumors, but also to a high treatment intensity in both groups. This demonstrates, that elderly LGG patients have tumor properties and treatment outcomes like younger patients and should encourage clinicians not to restrain from intensified treatment.

## Conclusion

This study presents a molecularly well-characterized, contemporary cohort of elderly lower-grade glioma patients. Younger and elderly patients conferred comparable surgical outcomes and PFS. Intensified treatment including maximal safe resection should therefore be advocated in elderly lower-grade glioma patients whenever feasible.

## Supplementary Information

Below is the link to the electronic supplementary material.

Supplementary file1 (DOCX 363 KB)
