# Supplementary material for: Isocitrate-dehydrogenase-mutant lower grade glioma in elderly patients: treatment and outcome in a molecularly characterized contemporary cohort
Source: J Neurooncol. 2023 Jan 17;161(3):605–15. doi: 10.1007/s11060-022-04230-1 (PMC9992027; doi:10.1007/s11060-022-04230-1)
Supplement: Supplementary file 1 — Supplementary file1 (DOCX 363 KB) [file 11060_2022_4230_MOESM1_ESM.docx]

# Supplementary Figures

## Supplementary Figure 1

**Suppl. Fig. 1:** Kaplan-Meier survival curves of tumor- and treatment- related factors vs. PFS in the younger and the elderly sub cohorts.

## Supplementary Figure 2

**Suppl. Fig. 2:** Comparison of Kaplan-Meier survival curves (overall survival) between the study cohort and “The Cancer Genome Atlas” (TCGA) IDH mutant WHO grade 2 and 3 study cohort. **a.** Overall survival was worse in the elderly in both study populations, although survival time in general was better in our cohort. **b.** In our study population WHO grade impacted only oligodendroglioma tumor subtype as compared to the TCGA cohort.

## Supplementary Figure 3

**Suppl. Fig. 3.:** Tumor subtype and age distritbution in „The Cancer Genome Atlas“ (TCGA) IDH mutant glioma WHO grade 2 and 3 study population. Results coincide very well with results of our study population. * p<0.05, *** p<0.001.
